# Erythropoietin prevents necrotizing enterocolitis in very preterm infants: a randomized controlled trial

**DOI:** 10.1186/s12967-020-02459-w

**Published:** 2020-08-08

**Authors:** Yong Wang, Juan Song, Huiqing Sun, Falin Xu, Kenan Li, Chunxia Nie, Xiaoli Zhang, Xirui Peng, Lei Xia, Ziyun Shen, Xiao Yuan, Shan Zhang, Xue Ding, Yaodong Zhang, Wenqing Kang, Liling Qian, Wenhao Zhou, Xiaoyang Wang, Xiuyong Cheng, Changlian Zhu

**Affiliations:** 1 Henan Key Laboratory of Child Brain Injury, Department of Neonatology, Institute of Neuroscience and Third Affiliated Hospital of Zhengzhou University, Zhengzhou, 450052 China; 2 Department of Neonatology, Children’s Hospital of Zhengzhou University, Zhengzhou, 450018 China; 3 Department of Neonatology, Women and Children Health Care Center of Luoyang, Luoyang, 471000 China; 4 Department of Neonatology, First Affiliated Hospital of Zhengzhou University, Zhengzhou, 450052 China; 5 Department of Pediatrics, Children’s Hospital of Fudan University, Shanghai, China; 6 Center of Perinatal Medicine and Health, Institute of Neuroscience and Physiology, Sahlgrenska Academy, University of Gothenburg, 40530 Gothenburg, Sweden; 7 Center for Brain Repair and Rehabilitation, Institute of Neuroscience and Physiology, Sahlgrenska Academy, University of Gothenburg, 40530 Gothenburg, Sweden; 8 Department of Women’s and Children’s Health, Karolinska Institutet, 17176 Stockholm, Sweden

**Keywords:** Preterm infant, Necrotizing enterocolitis, Erythropoietin

## Abstract

**Background:**

Necrotizing enterocolitis (NEC) is one of the most severe complications in very preterm infants, but there are currently no accepted methods to prevent NEC. Studies have shown that erythropoietin (EPO) has the potential to prevent NEC or improve outcomes of preterm NEC. This study aimed to determine whether recombinant human EPO (rhEPO) could protect against NEC in very preterm infants.

**Methods:**

The study was a prospective randomized clinical trial performed among four NICU centers. A total of 1327 preterm infants with gestational age ≤ 32 weeks were admitted to the centers, and 42 infants were excluded leaving 1285 eligible infants to be randomized to the rhEPO or control group. Infants in the rhEPO group were given 500 IU/kg rhEPO intravenously every other day for 2 weeks, while the control group was given the same volume of saline. The primary outcome was the incidence of NEC in very preterm infants at 36 weeks of corrected gestational age.

**Results:**

A total of 1285 infants were analyzed at 36 weeks of corrected age for the incidence of NEC. rhEPO treatment significantly decreased the incidence of NEC (stage I, II and III) (12.0% vs. 17.1%, *p *= 0.010), especially confirmed NEC (stage II and III) (3.0% vs. 5.4%, *p *= 0.027). Meanwhile, rhEPO treatment significantly reduced the number of red blood cells transfusion in the confirmed NEC cases (1.2 ± 0.4 vs. 2.7 ± 1.0, *p* = 0.004). Subgroup analyses showed that rhEPO treatment significantly decreased the incidence of confirmed NEC at gestational age < 28 weeks (*p *= 0.019), and the incidence of all stages NEC in preterm infants with hemoglobin < 90 g/l (*p* = 0.000) and 5 min Apgar score > 5 (*p* = 0.028).

**Conclusion:**

Repeated low-dose rhEPO treatment is beneficial against NEC in very preterm infants.

*Trial registration* The protocol was registered retrospectively at ClinicalTrials.gov (NCT03919500) on April 18, 2019. https://clinicaltrials.gov/ct2/show/NCT03919500

## Background

Necrotizing enterocolitis (NEC) is one of the most common causes of death in very preterm infants. The incidence of NEC is highly variable and is dependent on gestational age and birth weight, and it occurs in 5–22% of preterm infants with a birth weight < 1000 g [1]. Nearly one third of cases are fatal [2], and survivors are at high risk for poor long-term growth and neurodevelopmental disorders [3]. Despite preventive strategies including antenatal corticosteroid administration [4], breast milk feeding [5], probiotics [6], and lactoferrin supplementation [7], surgical treatment is still often inevitable. Therefore, strategies specifically for preventing and treating NEC are needed.

Recombinant human erythropoietin (rhEPO) is routinely used as an anti-anemia treatment in preterm infants in the NICU, and it also functions as an anti-inflammatory, antitoxin, and antioxidant [8]. Studies have reported that EPO treatment decreases the incidence and severity of experimental NEC in animal models [9–11], and a retrospective analysis showed that rhEPO (200 IU/kg intravenously with continuous infusion for 2 weeks starting on the first day of life, or 400 IU/kg subcutaneously three times a week) protected against NEC in very low birth weight infants [12]. Lower complication rates and reduced mortality were observed in NEC neonates receiving repeated low-dose rhEPO (200 IU/kg, intramuscular injection, twice a week for a total of 1 week) [13]. Enteral administration of rhEPO (88 IU/kg once daily until the enteral intake reached 100 ml/kg of milk, or after a maximum of 7 days) also decreased the risk of NEC in preterm neonates [14]. However, the optimum dose and timing of rhEPO treatment for NEC in very preterm infants is still uncertain and it could be varied personally because many factors can influence pharmacokinetics of rhEPO [15–17]. Our previous randomized controlled trial showed that repeated-dose rhEPO (500 IU/kg every other day for 2 weeks starting within 72 h after birth) promoted good long-term neurological outcomes in very preterm infants and also showed a protective tendency against NEC [18], but the total number of patients was not enough for accurately evaluating the effect of rhEPO against NEC. Thus, the aim of this study was to further investigate the effect of repeated low-dose rhEPO on NEC in very preterm infants.

## Methods

### Study design and participants

This was a prospective randomized clinical trial conducted in four NICU centers, including the Third Affiliated Hospital, Children’s Hospital, the First Affiliated Hospital of Zhengzhou University, and the Women and Children Health Care Center of Luoyang. Between January 2014 and June 2017, preterm infants with gestational age ≤ 32 weeks and hospitalized within 72 h after birth were deemed eligible for the study. Infants with genetic or metabolic diseases, congenital abnormalities, polycythemia, pneumothorax, grade III/IV intracranial hemorrhage, or unstable vital signs (such as respiration and circulation failure) before randomization were excluded from the study. Clinical information and complications were collected during hospitalization. Echocardiography and head ultrasound were examined routinely for all the infants, and hematological parameters were monitored every week. Anemia was defined as hemoglobin (Hb) ≤ 110 g/l, and red blood cell (RBC) transfusions were given for the infants with severe or moderate anemia with severe clinical manifestations [19]. Thrombocytopenia was defined as a platelet count of less than 100 × 10^9^/l [20], small for gestational age was defined as birth weight less than the 10th percentile for gestational age, and patent ductus arteriosus was defined as requiring pharmacological therapy or surgical ligation. All survivors were followed up for the incidence of NEC until 36 weeks of corrected gestational age. Written informed consent was obtained from the parents of all included infants. The trial is registered at ClinicalTrials (NCT03919500).

### Randomization and blinding

The preterm infants were randomly assigned to the rhEPO or control group by the trial investigators in a 1:1 allocation using a simple randomization plan by a computer-based random-number generator in each NICU center. The doctors and nurses in the NICU were aware of the patient treatment groups. The investigators performing quality control, data collection, and analysis were blinded to the treatment groups.

### Intervention

Infants in the rhEPO group were given rhEPO (500 IU/kg dissolved in 2 ml saline, intravenously once every other day for 2 weeks for a cumulative dose of 3500 IU/kg) starting within 72 h after birth. Infants in the control group were given saline with the same volume and timing. Other treatment and care of preterm infants followed the same routines in all four NICU centers. If the infants developed polycythemia, hypertension, thrombosis, shock, or any other emergency conditions, the rhEPO administration would be stopped immediately and appropriate emergency treatment would be given.

### Outcome

The primary outcome was the incidence of NEC in very preterm infants at 36 weeks of corrected gestational age. Clinical information, including gestational age, birth weight, sex, prenatal high-risk history, birth history, and complications during hospitalization, was collected by the investigators. Infants with suspected NEC (stage I), moderate NEC (stage II), or severe NEC (stage III) were categorized according to Bell’s classification standard [21]. In this study, confirmed NEC was defined as NEC stage II and III. Fulminant NEC was defined as NEC with rapid clinical progression with death or severe disease requiring surgical management occurring within 48 h of the onset of symptoms [22].

### Statistical analysis

Data were analyzed using SPSS 23.0 software (IBM, Armonk, NY). Student’s t-test and the chi-square test were used for baseline analysis. The chi-square test was used for group and subgroup analyses, and the Mantel–Haenszel test was used for interaction analyses in subgroups. A two-tailed *p*-value less than 0.05 was deemed significant.

The study was conducted in accordance with the Declaration of Helsinki, and the protocol was approved by the Ethics Committee of Zhengzhou University and Henan Medical Academy (201201002). The sample size was estimated based on the assumption that 12% of the very preterm infants in the NICU would develop confirmed NEC [23]. If the relative risk were to be decreased by 40% with rhEPO treatment, then 591 patients would need to be recruited for each group for a significance level of 5% with 80% power.

## Results

### Study population and baseline information

**Fig. 1:**
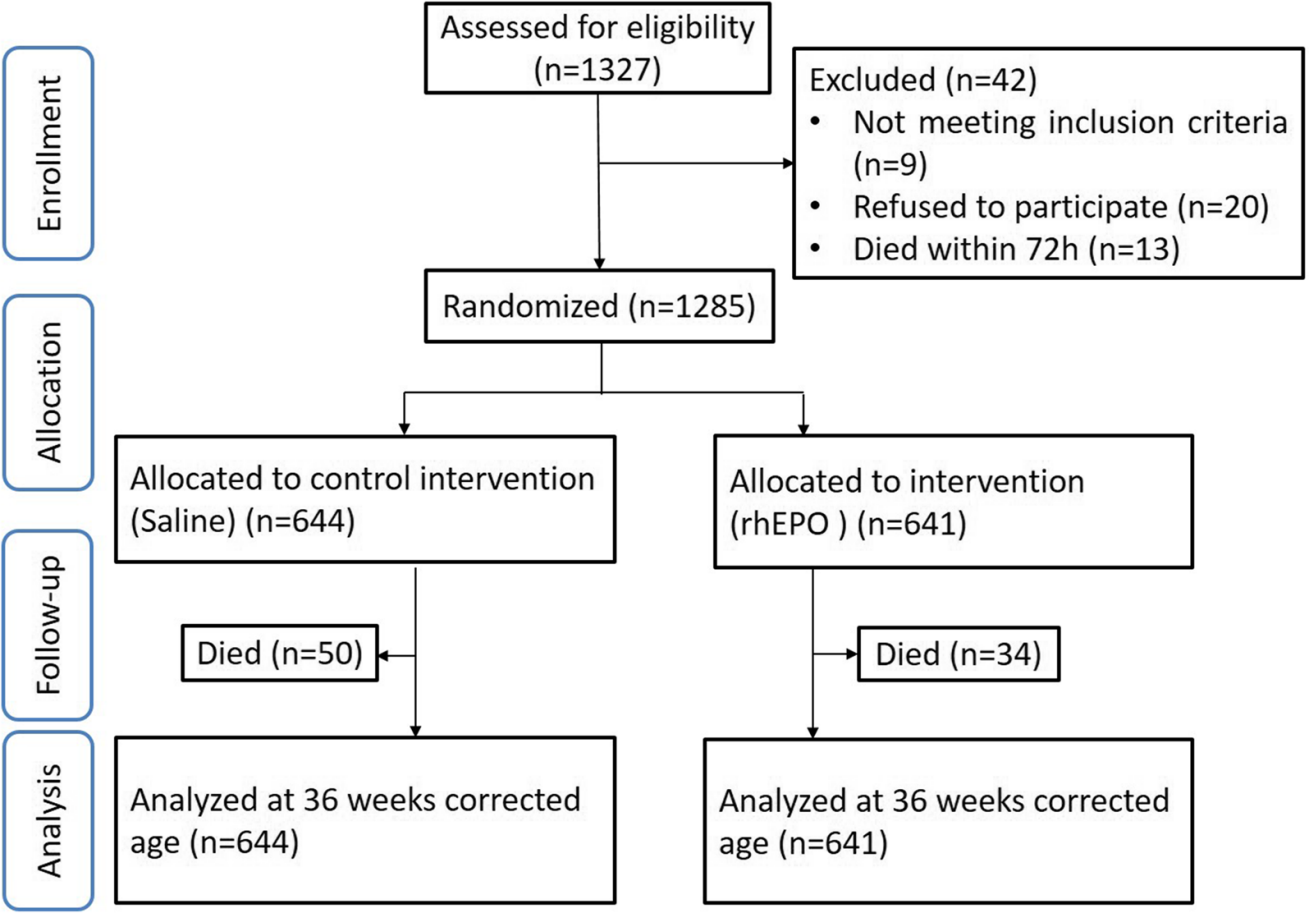
Study flow. Schematic flow chart showing the numbers of infants who were screened for eligibility, randomly assigned to rhEPO or placebo groups, and followed up to 18 months of corrected age

Between January 2014 and June 2017, a total of 1327 preterm infants with gestational age ≤ 32 weeks admitted to the four NICU centers were eligible. A total of 42 infants (3 with congenital megacolon, 3 with 21-trisomy, 3 with methylmalonic acidemia, 20 whose parents refused participation, and 13 who died within 72 h) were excluded, and 1285 infants were randomized to the rhEPO (641 infants) and control (644 infants) treatment groups. At 36 weeks of corrected gestational age (Fig. 1), a total 1285 infants were analyzed, and 84 infants died (35 died from respiratory failure, 34 died from sepsis, 8 died from pulmonary hemorrhage, 4 died from NEC, and 3 died from severe brain injury). There were no infants who developed polycythemia, hypertension, thrombosis, or emergency conditions in the rhEPO treatment group. There were no significant differences between the two groups for any of the baseline parameters (*p *> 0.05) (Table 1).

**Table 1:** Baseline information of very preterm infants

	Control n = 644	rhEPO n = 641	*p*-value	Total
Gestation age (weeks), median (quartile range)	30.0 (29.0–31.0)	29.7 (28.9–30.9)	0.138	29.9 (29.0–30.9)
Birth weight (g), median (quartile range)	1300 (1100–1450)	1250 (1100–1410)	0.131	1300 (1100–1450)
Small for gestational age (%)	132/644 (20.5)	149/641 (23.2)	0.228	281/1285 (21.9)
Male (%)	385/644 (59.8)	374/641(58.3)	0.601	759/1285 (59.1)
Severe asphyxia (%)	30/644 (4.7)	24/641 (3.7)	0.414	54/1285 (4.2)
Mechanical ventilation ≥ 7 days (%)	67/644 (10.4)	88/641 (13.7)	0.067	155/1285 (12.1)
Patent ductus arteriosus (%)	132/644 (20.5)	111/641 (17.3)	0.146	243/1285 (18.9)
Caesarean section (%)	384/644 (59.6)	401/641 (62.6)	0.281	785/1285 (61.1)
Premature rupture membranes (%)	234/644 (36.3)	227/641(35.4)	0.730	461/1285 (35.9)
Hypertension of pregnancy (%)	155/644 (24.1)	181/641 (28.2)	0.089	336/1285 (26.1)
First feeding (days), mean ± SD	4.3 ± 2.1	4.5 ± 2.2	0.324	4.4 ± 2.2
Formula milk feeding (%)	181/644 (28.1)	166/641 (25.9)	0.374	347/1285 (27.0)

### rhEPO treatment decreased the incidence of NEC in very preterm infants

A total of 51 infants (3.8%) of the 1285 very preterm infants developed confirmed NEC (Stage II and III). rhEPO treatment reduced the incidence of NEC (stage I, II and III) (17.1% vs. 12.0%, *p *= 0.010) and confirmed NEC (stage II and III) (5.4% vs. 3.0%, *p *= 0.027) compared to controls. However, rhEPO did not have a significant effect on NEC stage I (11.6% vs. 9.2%, *p *> 0.05), NEC stage II (3.9% vs. 2.3%, *p *> 0.05), or NEC stage III (1.6% vs. 0.6%, *p *> 0.05) (Table 2), or on fulminant NEC [5/110 (4.5%) in controls and 3/77 (3.9%) in the rhEPO group, p = 1.000]. No side effects were observed with rhEPO treatment. The average time to develop NEC was 11.9 ± 11.4 days after birth with a mean corrected gestational age of 31.4 ± 2.1 weeks. There was no significant effect on the time to develop NEC after birth (12.6 ± 11.5 days in control group vs. 10.7 ± 11.3 days in the rhEPO group, *p* = 0.555) or on the corrected gestational age at which NEC developed (31.4 ± 1.9 weeks in the control group vs. 31.5 ± 2.6 weeks in the rhEPO group, *p* = 0.864) (Table 3).

**Table 2:** rhEPO protected very preterm infants against NEC

NEC	Control n/total (%)	rhEPO n/total (%)	*p*-value	Total n/total (%)
Stage I	75/644 (11.6)	59/641 (9.2)	0.152	134/1285 (10.4)
Stage II	25/644 (3.9)	15/641 (2.3)	0.112	40/1285 (2.3)
Stage III	10/644 (1.6)	4/641 (0.6)	0.109	14/1285 (1.1)
Stage II and III	35/644 (5.4)	19/641 (3.0)	*0.027*	54/1285 (4.2)
Stage I, II and III	110/644 (17.1)	77/641 (12.0)	*0.010*	187/1285 (14.6)

**Table 3:** Anemia and thrombocytopenia in confirmed NEC

	Control n = 35	rhEPO n = 19	p-value	Total n = 54
Days at diagnosis with NEC, d	12.6 ± 11.5	10.7 ± 11.3	0.555	11.9 ± 11.4
Gestational age at diagnosis with NEC, w	31.4 ± 1.9	31.5 ± 2.6	0.864	31.4 ± 2.1
Anemia before NEC, n/total (%)	24/35 (68.6%)	11/19 (57.9%)	0.553	35/54 (64.8%)
Hb < 90 g/l before NEC, n/total (%)	8/35 (22.9%)	4/19 (21.1%)	0.879	12/54 (22.2%)
RBC transfusion before NEC, n/total (%)	8/35 (22.9%)	6/19 (31.6%)	0.528	14/54 (25.9%)
Time between transfusion and NEC, hours	29.8 ± 68.0	49.8 ± 89.7	0.361	36.8 ± 76.1
Number of RBC transfusions	2.7 ± 1.0	1.2 ± 0.4	*0.004*	2.0 ± 1.1
Thrombocytopenia, n/total (%)	15/35 (42.9)	10/19 (52.6)	0.573	25/54 (46.3)
Platelet count (× 10^9^/l)	135 ± 93	103 ± 73	0.204	123 ± 87

### Subgroup analysis of the effect of rhEPO on NEC in very preterm infants

There was no interaction effect of rhEPO against NEC (all stages) according to gestation age, birth weight, delivery mode, gender, mechanical ventilation, or 1 min Apgar score (interaction *p *> 0.05). However, rhEPO treatment significantly decreased the incidence of all stages NEC in preterm infants with Hb < 90 g/l (*p *< 0.0001, interaction p = 0.006) and with 5 min Apgar score > 5 (*p* = 0.028, interaction p = 0.012) (Table 4). Further analysis showed that the incidence of anemia before being diagnosed with NEC was no different between the control (24/35, 68.6%) and rhEPO (11/19, 57.9%, p = 0.553) groups. The ratio of packed RBC transfusion before NEC was not significantly different in the control (8/35, 22.9%) and rhEPO treatment group (6/19, 31.6%, p = 0.528), nor was the time from RBC transfusion to diagnosed NEC (29.8 ± 68.0 h in the control group and 49.8 ± 89.7 h in the rhEPO group; p = 0.361). However, rhEPO treatment significantly decreased the number of RBC transfusion in confirmed NEC (2.7 ± 1.0 in control vs. 1.2 ± 0.4 in rhEPO group, *p* = 0.004). The platelet count in the diagnosed NEC infants was 135 ± 93 × 10^9^/l in the control group and 103 ± 73 × 10^9^/l in the rhEPO group (p = 0.204), and the incidence of thrombocytopenia was also not significantly different between controls (15/35, 42.9%) and the rhEPO treatment group (10/19, 52.6%, p = 0.573) (Table 3). Furthermore, the effect of rhEPO against confirmed NEC (stage II and III) was observed in preterm infants with gestation age < 28w (*p *< 0.05) with an interaction *p*-value of 0.013, but not for the infants with gestation age 28–29^6/7^w or 30–32w, (*p *> 0.05). There were no interaction effect on rhEPO against confirmed NEC of different birth weights, delivery modes, mechanical ventilation, serum hemoglobin, sex, or Apgar scores at 1 min and 5 min (interaction *p *> 0.05 for all) (Table 5).

**Table 4:** Subgroup analysis the effect of rhEPO on all stages NEC

	Control n/total (%)	rhEPO n/total (%)	*p*-value	Relative risk [95% CI]	Interaction *p*	Total n/total (%)
Gestation age						
< 28w	11/53 (20.8)	8/57 (14.0)	0.352	0.623 [0.229–1.694]		19/110 (17.3)
28–29^6/7^w	49/258 (19.0)	33/288 (11.5)	*0.014*	0.552 [0.342–0.890]		82/546 (15,0)
30–32w	50/333 (15.0)	36/296 (12.2)	0.299	0.784 [0.495–1.242]	0.581	86/629 (13.7)
Birth weight						
< 1000 g	18/54 (33.3)	5/50 (10.0)	*0.004*	0.222 [0.075–0.657]		23/104 (22.1)
1000–1499 g	70/432 (16.2)	54/462 (11.7)	0.051	0.684 [0.467–1.003]		124/894 (13.9)
≥ 1500 g	22/158 (13.9)	18/129 (14.0)	0.994	1.002 [0.512–1.962]	0.060	40/287 (13.9)
Delivery mode						
Vaginal delivery	39/260 (15.0)	26/240 (1.7)	0.166	0.688 [0.405–1.170]		65/500 (13.0)
Caesarean	71/384 (18.5)	51/401 (12.7)	*0.026*	0.642 [0.435–0.949]	0.837	122/785 (15.5)
Gender						
Girl	41/259 (15.8)	27/267 (10.1)	0.051	0.598 [0.356–1.051]		68/526 (12.9)
Boy	69/385 (17.9)	50/374 (13.4)	0.085	0.707 [0.476–1.050]	0.616	119/759 (15.7)
Mechanical ventilation						
< 7 days	85/577 (14.7)	62/553 (11.2)	0.079	0.731 [0.515–1.038]		147/1130 (13.0)
≥ 7 days	25/67 (37.3)	15/88 (17.0)	*0.004*	0.345 [0.164–0.727]	0.072	40/155 (25.8)
1 min Apgar score						
> 3	105/614 (17.1)	76/617 (12.3)	*0.018*	0.681 [0.495–0.937]		181/1231 (14.7)
≤ 3	5/30 (16.7)	1/24 (4.2)	0.146	0.217 [0.024–2.003]	0.299	6/54 (11.1)
5 min Apgar score						
> 5	104/622 (17.3)	74/608 (12.2)	*0.028*	0.690 [0.500–0.952]		178/1230 (14.5)
≤ 5	6/22 (27.3)	3/33 (9.1)	0.134	0.267 [0.059–1.211]	*0.012*	9/55 (16.4)
Hemoglobin						
≥ 90 g/l	31/296 (10.5)	36/301 (12,0)	0.565	1.161 [0.698–1.933]		67/597 (11.2)
< 90 g/l	79/348 (22.7)	41/340 (12.1)	*0.000*	0.467 [0.309–0.705]	*0.006*	120/688 (17.4)

**Table 5:** Subgroup analysis of the effect of rhEPO on confirmed NEC

	Control n/total (%)	rhEPO n/total (%)	*p*-value	Relative risk [95% CI]	Interaction *p*	Total n/total (%)
Gestation age						
**< **28 w	9/53 (17.0)	2/57 (3.5)	*0.019*	0.178 [0.037–0.865]		11/110 (10.0)
28–29^6/7^ w	14/258 (5.4)	7/288 (2.4)	0.069	0.434 [0.172–1.093]		21/546 (3.8)
30–32 w	12/333 (3.6)	10/296 (3.4)	0.878	0.935 [0.398–2.198]	*0.013*	22/629 (3.5)
Birth weight						
< 1000 g	12/54 (22.2)	2/50 (4.0)	*0.007*	0.146 [0.031–0.689]		14/104 (13.5)
1000–1499 g	19/432 (4.4)	10/462 (2.2)	0.060	0.481 [0.221–1.046]		29/894 (3.2)
≥ 1500 g	4/158 (2.5)	7/129 (5.4)	0.204	2.209 [0.632–7.720]	0.142	11/287 (3.8)
Delivery mode						
Vaginal delivery	15/260 (5.8)	4/240 (1.7)	*0.017*	0.277 [0.091–0.846]		19/500 (3.8)
Caesarean	20/384 (5.2)	15/401 (3.7)	0.319	0.707 [0.357–1.402]	0.154	35/785 (4.5)
Gender						
Girl	12/259 (4.6)	4/267 (1.5)	*0.036*	0.343 [0.108–1.091]		16/526 (3.0)
Boy	23/385 (6.0)	15/374 (4.0)	0.215	0.647 [0.332–1.260]	0.267	38/759 (5.0)
Mechanical ventilation						
< 7 days	28/577 (4.9)	17/553 (3.1)	0.126	0.622 [0.336–1.149]		45/1130 (4.0)
≥ 7 days	7/67 (10.4)	2/88 (2.3)	*0.031*	0.199 [0.040–0.993]	0.181	9/155 (5.8)
1 min Apgar score						
> 3	32/614 (5.2)	19/617 (3.1)	0.061	0.578 [0.324–1.031]		51/1231 (4.1)
≤ 3	3/30 (10.0)	0/24 (0.0)	0.111	0.900 [0.799–1.014]	0.226	3/54 (5.6)
5 min Apgar score						
> 5	34/622 (5.5)	19/608 (3.1)	0.060	0.558 [0.315–0.989]		53/1230 (4.3)
≤ 5	1/22 (4.5)	0/33 (0.0)	0.400	0.389 [0.278–0.543]	0.053	1/55 (1.8)
Hemoglobin						
≥ 90 g/l	11/296 (3.7)	7/301 (2.3)	0.320	0.617 [0.236–1.614]		18/597 (3.0)
< 90 g/l	24/348 (6.9)	12/340 (3.5)	*0.047*	0.494 [0.243–1.004]	0.715	36/688 (5.2)

Confirmed NEC means NEC stage II and III

## Discussion

NEC is one of the most severe complications in preterm neonates and is associated with high morbidity and mortality. NEC often occurs in outbreaks, suggesting the involvement of infectious agents [24, 25], and it proceeds through a cascade of events before any effective treatment can be applied. Therefore, the development of preventive strategies against NEC is of utmost importance. This prospective multi-center randomized controlled trial was an extension of our previous study [18]. Here we found that repeated low-dose rhEPO treatment (500 IU/kg) significantly decreased the incidence of confirmed NEC. Thus the results observed in the current study are in line with the results of animal models of NEC [9–11] and the results of other clinical studies [12–14], all using different doses of rhEPO and different routes of administration. Previous prospective clinical studies have all been hampered by small numbers of patients [13, 14], and the current study had a larger sample size than all similar previous studies of preterm infants. The results of this study strongly suggest that rhEPO is a potential promising therapeutic target for the prevention and/or treatment of NEC in very preterm infants.

Erythropoietin receptors have been observed on enterocytes in the developing human fetus and in human neonates [26, 27], and based on all of the work described below we speculate that rhEPO plays an important role against NEC in very preterm infants through its anti-inflammatory actions, by maintaining the integrity of the intestinal barrier, by inhibiting apoptosis, and by inhibiting oxidative stress. Animal models of NEC have suggested that EPO improves NEC injury by reducing inflammatory reactions [28] and by maintaining the integrity of the intestinal barrier by preventing the loss of the tight junction protein ZO-1 and by normalizing other intestinal epithelial tight junction components [10]. In addition, EPO decreases both autophagy and apoptosis via the Akt/mTOR signaling pathway and the MAPK/ERK pathway, and thus it reduces intestinal mucosa injury from inflammation [11]. Studies also indicate that EPO reduces the occurrence of NEC in mice and rats by inhibiting the lipid peroxidation mediated by oxygen-free radicals [29] and the excessive production of nitric oxide [9, 30, 31], both of which are associated with the pathogenesis of NEC. In infants, rhEPO (200 IU/kg twice a week for a total of 1 week) reduced neonatal NEC injury by reducing inflammation [13]. Notably, a recent study reported that relatively high endogenous neonatal EPO concentrations in the blood are associated with increased risk of NEC requiring surgery [32], which seems to be in conflict with our results. However, such relatively increased endogenous EPO levels might be due to severe stress status in the infants leading to the stimulation of endogenous EPO production for self-protection, but the increased endogenous EPO levels are still too low to have a sufficiently protective effect to avoid surgery in these infants. Indeed, a recent study showed that endogenous EPO levels in the infants of the control group were very low compared to the concentrations resulting from exogenous rhEPO administration in the experimental group and in whom a protective effect was achieved [33]. Therefore, relatively high endogenous EPO levels in preterm infants might indicate a potentially severe status and that exogenous rhEPO administration is needed.

Subgroup analyses showed that rhEPO treatment significantly decreased the incidence of all stages NEC in preterm infants with Hb < 90 g/l. Multiple observational studies have reported an association between severe anemia and the development of NEC [34–36]. Anemia can impair splanchnic perfusion and increase intestinal inflammation and barrier disruption leading to tissue hypoxia and anaerobic metabolism and thus predisposing to ischemic injury and possibly to NEC [37–40]. Thus, preventing anemia could be a strategy to prevent the development of NEC, and RBC transfusion is a commonly used method to achieve this [41, 42]. However, other studies and meta-analyses showed an association between RBC transfusion and increased risk of NEC [40, 43, 44]. The conflicting data regarding the role of anemia and RBC transfusion on the development of NEC remains unclear. Our data show that rhEPO could reduce the number of RBC transfusion in confirmed NEC, and that rhEPO could be beneficial for preterm infants against NEC, especially in those with Hb < 90 g/l. Asphyxia is widely accepted as a risk factor for NEC. Hypoxia-reoxygenation could induce NEC in animal model [45]. Evidences show that EPO protects against NEC in newborn rats model induced by hypoxia-reoxygenation [10, 11, 38]. However, how does the severity of asphyxia affect the protective effect of EPO is unclear. In our study, rhEPO treatment significantly decreased the incidence of all stages NEC in preterm infants with 5 min Apgar score > 5, which indicates that severe asphyxia might induce irreversible damage to intestinal tract that beyond the preventive and therapeutic ability of rhEPO treatment.

Meanwhile, we found that rhEPO treatment significantly decreased the incidence of confirmed NEC (stage II and III) at a gestational age of < 28w. It is reported that susceptibility to NEC is inversely related to gestational age [46, 47], and the immature intestinal barrier in lower gestational age infants might impact the effect of rhEPO therapy. rhEPO is also a neuroprotective drug, and its use in preterm infants has been heavily discussed in recent years [30]. Recently, one study [48] reported that there was no effect of rhEPO treatment (1000 U/kg for 6 doses after birth, then 400 U/kg three times per week up to 32 weeks of postmenstrual age) against NEC (all stages) in preterm infants of less than 28 weeks of gestational age. Similarly, we did not find a protective effect of rhEPO against all stages NEC in infants of less than 28 weeks of gestational age, but we did see an effect in infants of 28–29^6/7^ weeks of gestational age. However, our subgroup analysis showed that rhEPO has a preventive effect on the incidence of confirmed NEC in the infants of less than 28 weeks of gestational age, although the sample size of infants less than 28 weeks of gestational age in our study was much less than theirs (110 vs. 741 infants), but no subgroup analysis was available for the confirmed NEC cases from the published report [48]. Meanwhile, the high incidence of retinopathy of prematurity (ROP) (51% in the EPO group vs. 56% in the control group) in the published study indicates that the studied population or treatment protocol for the very preterm infants might have influenced the incidence of preterm complications and the therapeutic effect of rhEPO. A further study focusing on preterm infants of less than 28 weeks of gestational age as well as the effect of EPO on ROP is being performed (NCT02745990). Our current findings and the growing body of research on rhEPO suggest that rhEPO might be a promising drug for multiple complications in very preterm infants.

rhEPO was originally used at a low dose to prevent anemia in preterm infants, and it is now widely accepted as a routine application for all preterm infants as a substitute for blood transfusion [49–51]. Recent studies showed that rhEPO has multiple beneficial effects on preterm infants [18, 52, 53]. Our previous study with repeated administration of as low as 500 U/kg of rhEPO for 2 weeks showed not only neuroprotection, but also a reduced incidence of NEC and sepsis, although the mechanisms behind this are not fully understood [18]. In this study, even though rhEPO treatment reduced RBC transfusion and incidence of NEC, it did not do so in all of the preterm infants. This could be related to interpatient variability in rhEPO responsiveness, and a pharmacokinetics approach might be an option to optimize rhEPO treatment in preterm infants [15, 16].

There were some shortcomings in our study that need to be mentioned. First, the diagnosis of stage I NEC was not easily confirmed and was often confounded with feeding intolerance, we did not consider this in our primary outcome. Although there are several categories of biomarkers for NEC [54] such as inflammatory cytokines, gut-associated proteins, and microRNAs, none of them are specific for NEC. Second, exclusive breastfeeding has not yet been implemented in the four NICUs of our study, which might have affected the incidence of NEC to some extent. Third, the small sample size of confirmed NEC patients restricted the evaluation of long-term outcomes. Further data collection and follow-up to 1–2 years are ongoing. Fourth, the dose and treatment protocol of rhEPO was based on our previous study without further optimization according to pharmacokinetics/pharmacodynamics data in the study population.

## Conclusions

To our knowledge, this is the first large-sample prospective study showing that low-dose rhEPO prevents NEC in very preterm infants with gestational age ≤ 32 weeks. Repeated low-dose rhEPO treatment reduced the risk of NEC compared with controls and without obvious adverse effects. This indicates that prophylactic early rhEPO treatment has multiple beneficial effects in very preterm infants and that rhEPO could be recommended for infants at risk of NEC. Whether such treatment improves long-term outcomes of NEC patients remains to be determined.

## Data Availability

The datasets used and/or analyzed during the current study are included in this published article.

## Abbreviations

Hb: Hemoglobin
NEC: Necrotizing enterocolitis
NICU: Neonatal intensive care unit
rhEPO: Recombinant human erythropoietin
RBC: Red blood cell
